# Chronic oral mucocutaneous candidiasis, recurrent respiratory infection, hepatosplenomegaly, and autoimmune diabetes mellitus: A case report of a gain-of-function mutation of *STAT1* in a Chinese boy

**DOI:** 10.3389/fped.2022.1001290

**Published:** 2022-10-11

**Authors:** Bingyan Cao, Meijuan Liu, Yun Zhao, Chunxiu Gong

**Affiliations:** ^1^ Department of Endocrinology, Genetic and Metabolism, Beijing Children’s Hospital, Capital Medical University, National Center for Children’s Health, Beijing, China; ^2^ Department of Pediatric, Second Hospital of Shijiazhuang, Shijiazhuang, China

**Keywords:** STAT1, chronic mucosal candidiasis, gain-of-function, diabetes mellitus, case report

## Abstract

**Background:**

Signal transducer and activator of transcription 1 (STAT1) gain-of-function (GOF) mutations are characterized by chronic mucocutaneous candidiasis and autoimmune diseases. Type 1 diabetes mellitus is one of the well-characterized autoimmune conditions.

**Case presentation:**

We reported a 5-year-old boy who presented with polydipsia and polyuria, with a medical history of chronic oral mucocutaneous candidiasis, recurrent respiratory infection, hepatosplenomegaly, and abnormal liver function. Genetic analysis identified a heterozygous GOF mutation (c.866A > G, p.Y289C) in *STAT1*.

**Results:**

Various medicines were given to the boy during the follow-up, including insulin to keep blood glucose stable, intravenous immunoglobulin and antifungal agents for recurrent infections, and antituberculosis drugs (isoniazid, rifampicin) to combat tuberculosis infection. He did not show recurrent infection, but chronic oral mucocutaneous candidiasis still occurred twice per month. The blood glucose level was well controlled.

**Conclusion:**

This article illustrates that early diagnosis and identification of *STAT1* mutation are essential for assessing the severity of the disease and determining reasonable treatment options.

## Background

The signal transducer and activator of transcription 1 (STAT1) is one of the seven members of STATs. STATs transmit cell signals from extracellular to intracellular through Janus kinases (JAKs), activate target genes, and regulate cell growth and differentiation (1, 2). Mutations in the gene encoding the STAT protein family are associated with immune dysregulation syndrome of autoimmune deficiency (3). STAT1 can promote apoptosis and inhibit cell growth and differentiation. There are four types of primary immunodeficiency diseases caused by *STAT1* mutation, including autosomal dominant gain-of-function (GOF) immunodeficiency, loss-of-function immunodeficiency, and autosomal recessive partial and complete immunodeficiency. Among these, the autosomal dominant inherited *STAT1-*GOF immunodeficiency is mainly associated with chronic mucosal candidiasis (CMC) and autoimmune diseases (3). As an autoimmune manifestation, diabetes mellitus is less common than hypothyroidism, and it is often diagnosed as type 1 diabetes mellitus (T1DM) at its onset. It is necessary to perform a genetic analysis to accurately diagnose monogenic autoimmune disease. In cases in which a clinical phenotype contains multiple system disorders, a genetic etiology may be indicated. Individuals suffering from autoimmunity due to a single gene defect may benefit from personalized treatment.

This report describes a boy with multiple system disorders who was identified with a *STAT1-*GOF mutation through whole exon sequencing (WES), and the prognosis was followed.

## Case presentation

### Patient information

A 5 year and 4 month old boy was referred to us for polydipsia and polyuria. His blood glucose was 29.5 mmol/L, hemoglobin A1c (HbA1c) was 8.9%, and fasting C-peptide was 0.07 ng/ml. Serum anti-glutamate decarboxylase antibody and anti-islet cell antibody were both positive. He was diagnosed with T1DM at the onset. He had a history of recurrent oral mucocutaneous Candida fungi and respiratory infections after birth. At the age of 1 year, he suffered from herpetic pharyngitis with alanine transferase (ALT) 1,000–2,000 U/L, which was relieved by anti-infective and liver-protecting treatment. Increased ALT levels occurred several times after infection. A left under axillary abscess incision and drainage was performed when he was 3 years and 8 months old. He was the first child of non-consanguineous parents without any family history of hereditary diseases.

### Clinical findings

Physical examination showed the weight was 18 kg (25th percentile), and the height was 110 cm (25th percentile). The blood pressure was normal. He was conscious, his thyroid and cardiopulmonary systems were normal, and there was no evidence of an enlarged lymph node. The liver is 4 cm under the right rib, and the spleen is 5 cm below the left rib, with blunt edges, and there is no swelling in the lower limbs.

Laboratory investigation revealed normal blood routine analysis, thyroid function, immunoglobulin A (IgA), immunoglobulin M, immunoglobulin G, ammonia, lactic acid, blood amino acids, acylcarnitine, and urine organic acid analysis. The level of aspartate aminotransferase was 100 U/L, ALT was 16.2 U/L. Hepatophilic virus antibodies [anti-cytomegalovirus-immunoglobulin M (CMV-IgM), anti-Epstein–Barr virus (EBV)-IgM, hepatitis A/B/C/E virus antibodies] and autoimmune liver disease antibodies were negative. The thyroglobulin antibody, thyroid peroxidase antibody, and thyroid-stimulating hormone receptor antibody were negative. The percentage of CD4 lymphocytes was 21%, CD8 lymphocytes was 45%, and CD4/CD8 was 0.46, which indicated immunodeficiency, the detailed laboratory information was shown in Table 1.

**Table 1:** Clinical characteristics of the patient in our present study.

	Results	Unit	Reference intervals
First time referred to our hospital			
Age	Five years and four months	—	—
Sex	Male	—	—
Height	110	cm	P25
Weight	18	kg	P25
Localization of candidiasis	Oral mucocutaneous Candida fungi	—	—
Autoimmunity	Type 1 diabetes mellitus autoimmune hepatitis at 1 year old	—	—
Infection	Respiratory infection three times/year from birth, axillary abscess at 3 years and 8 months old	—	—
ALT	16.2	U/L	5–40
AST	100	U/L	5–40
White blood cell count	5.69	10^9^/L	4.0–10.0
Neutrophil count	2.89	10^9^/L	1.4–6.5
Eosinophil count	0.09	10^9^/L	0.05–0.50
Lymphocyte count	2.24	10^9^/L	0.92–5.3
Helper T-cell CD4	21.0	%	25.0–57.0
Suppressor/cytotoxic T-cell CD8	45.0	%	14.0–34.0
CD4/CD8 ratio	0.46	—	1.1–2.0
Natural killer cell	7.4	%	7.0–40.0
IgG level	11.6	g/L	4.28–21.9
IgM level	0.62	g/L	0.48–2.26
IgA level	1.87	g/L	0.44–0.41
HbA1c	8.2	%	4.0–6.0
C-peptide	0.07	ng/ml	1.1–5.0
GAD	Positive	—	negative
Antibodies of autoimmune liver disease	Negative	—	negative
Thyroglobulin antibody	Negative	—	negative
Thyroid peroxidase antibody	Negative	—	negative
Thyroid-stimulating hormone receptor antibody	Negative	—	negative
Last follow-up			
Age	8 years old	—	—
Height	128	cm	P25–50
Weight	27	kg	P50
Localization of candidiasis	Oral mucocutaneous Candida fungi twice per month	—	—
Infection	Tuberculosis infection	—	—
HbA1c	6.5	%	4.0–6.0

ALT, alanine transferase; AST, aspartate aminotransferase; HbA1c, hemoglobin A1c; Ig, immunoglobulin; GAD, glutamic acid decarboxylase.

### Diagnostic assessment

**Figure 1:**
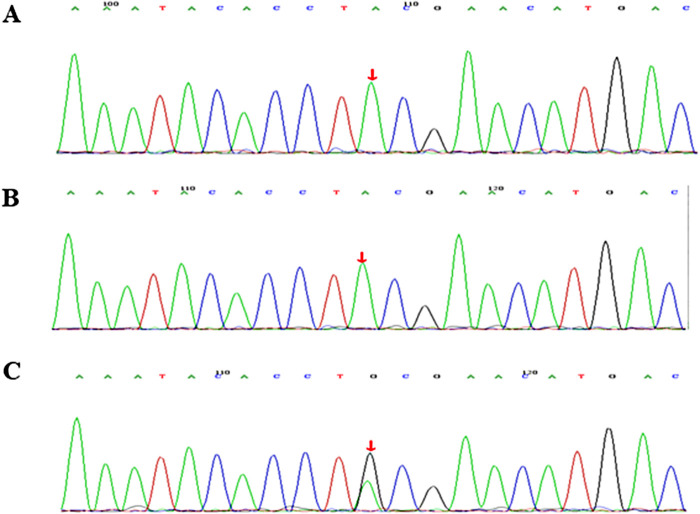
Molecular characterization of a family with STAT1 gain-of-function mutation. Sequence chromatogram showing a missense variant c.866A > G, p.Y289C. The variant was heterozygous in the proband (**C**). Sanger sequencing results of his father (**A**) and mother (**B**).

WES identified a heterozygous *de novo STAT1* mutation in the child, c.866A > G, p.Y289C, which was further confirmed by Sanger sequencing (Figure 1). It was previously shown to be a GOF mutation (4) and was defined as pathogenic according to criteria published by the American College of Medical Genetics and Genomics (ACMG) (5). The child was corrected diagnosed with monogenic autoimmune diabetes.

Based on the presence of CMC, immunodeficiency, autoimmune diabetes, and autoimmune hepatitis, as well as genetic results, the boy was diagnosed with syndromic CMC that was caused by a heterozygous GOF mutation of *STAT1*.

### Therapeutic intervention

Upon admission, he was given rapid-acting insulin with meals and long-acting insulin at bedtime, with a total amount of 1.27 IU/kg/d, with blood glucose levels of 6–7 mmol/L before meals and 6–12 mmol/L after meals. During the follow-up, he was given oral antifungal therapy of fluconazole (75 mg/d) and intravenous injection of human immunoglobulin once a month for candidiasis and recurrent infections.

### Follow-up and outcomes

As of now, he was 8 years old and his insulin dosage was 1.1 IU/kg/d. Even under fluconazole therapy, he developed oral mucocutaneous Candida fungi twice every month, but he did not develop recurrent viral or bacterial infections. He was given antituberculosis drugs (isoniazide, rifampicin) for tuberculosis infection. The liver function was normal at the last follow-up, and the HbA1c was 6.5%. Ruxolitinib was recommended to the patient’s parents, but they refused because of concerns about side effects.

## Discussion

The mutations of *STAT1* increase STAT1 phosphorylation by impairing nuclear dephosphorylation, which enhance signaling downstream of cytokines interferon α/β (IFN-α/β), IFN-γ, interleukin-27 (IL-27), STAT3-dependent IL-6 and IL-21 (6–9), and impair IL-17A, IL-17F immunity, resulting in lower T-cell counts producing IL-17A and/or IL-17F. CMC is most likely the consequence of impaired IL-17A and IL-17F immunity (10). Autoimmunity appears to be a consequence of stronger IFN-α/β signaling. The mutation reported in our study is located in the coiled-coil domain (CCD), which resulted in persistent STAT1 phosphorylation without appropriate dephosphorylation in all-natural killer cells in response to IFN-α (4).

More than one-third of patients with *STAT1*-GOF mutations presented with autoimmune manifestations. Autoimmunity predominantly affected endocrine organs, hypothyroidism was the most common endocrine disease, followed by diabetes mellitus and hyperthyroidism. Other autoimmune diseases contained hematological autoimmunity (hemolytic anemia or autoimmune thrombocytopenia, and autoantibody-positive pernicious anemia), systemic lupus erythematosus, scleroderma, autoimmune hepatitis, cutaneous diseases (vitiligo, alopecia, psoriasis), celiac disease, and inflammatory bowel disease (Crohn’s disease, ulcerative colitis, enteropathy with lymphocytic infiltration) (11). There have also been cases of patients with severe autoimmune symptoms resembling those found in patients with immune dysregulation-polyendocrinopathy-enteropathy-X-linked (IPEX) syndrome (12). Most patients with autoimmune manifestations were positive for autoantibodies.

The reason for diabetes mellitus here is different from that of T1DM. While both of them are autoimmune diseases with the associated islet autoantibodies, there is a crucial difference in their genetic susceptibility and hence their cause. The former is caused by a variant in a single gene in a monogenic autoimmune form of diabetes and is not associated with human leukocyte antigen (HLA). However, T1DM is a polygenic autoimmune disease with well-described associations with common variants in the HLA locus.

To date, over 20 cases of diabetes with a GOF mutation of *STAT1* have been reported in the literature (11, 12–21): 11 females and 9 males. In 11 cases, the onset age was less than 1 year old. The clinical manifestations at the onset were thrush, skin esophageal/genital mucosa infections caused by *Candida albicans*, repeatedly bacterial infections (*Staphylococcus aureus*, *Streptococcus pneumonia*, *Pseudomonas aeruginosa*, *Haemophilus influenza*, etc.), virus (herpes simplex virus, varicella-zoster virus, CMV, EBV, infectious wart) infections caused pneumonia, sepsis, etc., or with invasive fungal infections, such as fungal pneumonia and esophagitis. Hepatosplenomegaly or autoimmune hepatitis occurred in seven cases, hypothyroidism, thrombocytopenia, and autoimmune hemolytic anemia occurred in six cases. Of the 20 patients, 4 died of severe infection, 2 died of intracranial hemorrhage, and 1 died of complications after transplantation. The oldest surviving person was 59 years old. Before 5 years old, T lymphocytes and immunoglobulin were normal. T lymphocyte and immunoglobulin gradually decreased with age, and significantly decreased during puberty.

The outcomes of patients with *STAT1-*GOF mutations were poor. It was estimated that 12.04% of patients with *STAT1-*GOF mutations died prematurely, which were caused by severe infections (i.e., coccidioidomycosis, cytomegalovirus, septicemia), cancers, and cerebral hemorrhage due to aneurysms (10). A retrospective study of 15 patients with severe and life-threatening infections who received hematopoietic stem cell transplantation found that 6 patients survived, 4 died of systemic severe infections, 4 died of multiorgan failure after transplantation, and 1 patient died of a ruptured aneurysm (14).

It has been reported that the granulocyte colony-stimulating factor, JAKs inhibitor, and histone deacetylase inhibitor can be used to treat chronic mucosal candidiasis and alleviate clinical symptoms. Ruxolitinib, a JAKs inhibitor, can decrease the level of phosphorylation of STAT1 (22), improving the function of damaged Th17 cells, increasing the expression of IL-17, reducing IFN-gamma, and alleviating autoimmune reactions. A diabetic patient who stopped insulin therapy under ruxolitinib medication was in good control of his blood sugar 1 year later (23). In spite of this, further research is needed to assess ruxolitinib’s therapeutic effects. In addition, long-term intravenous immunoglobulin and antifungal therapy have also been reported to be effective (22). The child in our study received intravenous immunoglobulin infusion and antifungal therapy, he did not show recurrent infection, but CMC still occurred twice per month. Meanwhile, the child did not develop other autoimmune diseases such as hypothyroidism or thrombocytopenia. Ruxolitinib was not tried on the child because of the parents’ unwillingness, insulin was given to keep glucose under good control.

In conclusion, *STAT1* sequence analysis should be performed as soon as possible in patients suffering from recurrent respiratory infections and chronic mucosal candidiasis combined with autoimmune. Early diagnosis and identification of the type of *STAT1* mutations are important for assessing the severity of the disease and determining the appropriate treatment.

## Data Availability

The original contributions presented in the study are included in the article/Supplementary Material, further inquiries can be directed to the corresponding author.
